# *Propionibacterium freudenreichii* Surface Protein SlpB Is Involved in Adhesion to Intestinal HT-29 Cells

**DOI:** 10.3389/fmicb.2017.01033

**Published:** 2017-06-08

**Authors:** Fillipe L. R. do Carmo, Houem Rabah, Song Huang, Floriane Gaucher, Martine Deplanche, Stéphanie Dutertre, Julien Jardin, Yves Le Loir, Vasco Azevedo, Gwénaël Jan

**Affiliations:** ^1^Federal University of Minas Gerais – Instituto de Ciências BiológicasBelo Horizonte, Brazil; ^2^Science et Technologie du Lait et de l’Oeuf, Institut National de la Recherche Agronomique, Agrocampus OuestRennes, France; ^3^Pôle Agronomique OuestRennes, France; ^4^Suzhou Key Laboratory of Green Chemical Engineering, School of Chemical and Environmental Engineering, College of Chemistry, Chemical Engineering and Material Science, Soochow UniversitySuzhou, China; ^5^Microscopy Rennes Imaging Center, Biosit – UMS CNRS 3480/US, INSERM 018, University of Rennes 1Rennes, France

**Keywords:** adhesion, immunomodulation, surface proteins, probiotic, SlpB

## Abstract

*Propionibacterium freudenreichii* is a beneficial bacterium traditionally used as a cheese ripening starter and more recently for its probiotic abilities based on the release of beneficial metabolites. In addition to these metabolites (short-chain fatty acids, vitamins, and bifidogenic factor), *P. freudenreichii* revealed an immunomodulatory effect confirmed *in vivo* by the ability to protect mice from induced acute colitis. This effect is, however, highly strain-dependent. Local action of metabolites and of immunomodulatory molecules is favored by the ability of probiotics to adhere to the host cells. This property depends on key surface compounds, still poorly characterized in propionibacteria. In the present study, we showed different adhesion rates to cultured human intestinal cells, among strains of *P. freudenreichii*. The most adhesive one was *P. freudenreichii* CIRM-BIA 129, which is known to expose surface-layer proteins. We evidenced here the involvement of these proteins in adhesion to cultured human colon cells. We then aimed at deciphering the mechanisms involved in adhesion. Adhesion was inhibited by antibodies raised against SlpB, one of the surface-layer proteins in *P. freudenreichii* CIRM-BIA 129. Inactivation of the corresponding gene suppressed adhesion, further evidencing the key role of *slpB* product in cell adhesion. This work confirms the various functions fulfilled by surface-layer proteins, including probiotic/host interactions. It opens new perspectives for the understanding of probiotic determinants in propionibacteria, and for the selection of the most efficient strains within the *P. freudenreichii* species.

## Introduction

*Propionibacterium freudenreichii* is a GRAS (Generally Recognized As Safe) actinobacterium consumed in high amounts in fermented dairy products. It is a beneficial bacterium used in the food industry for the production of vitamins, for cheese ripening, and for its probiotic properties (Cousin et al., 2010). Probiotics are defined as “living microorganisms which when administered in adequate amounts confer a health benefit on the host” (Food and Agriculture Organization of the United Nations and World Health Organization, 2002). *P. freudenreichii* indeed revealed probiotic traits including modulation of intestinal inflammation (Mitsuyama et al., 2007; Foligné et al., 2010, 2013), as well as properties linked to the production of beneficial metabolites such as short-chain fatty acids (Jan et al., 2002; Lan et al., 2007; Cousin et al., 2012b), vitamins and the bifidogenic compound 1,4-dihydroxy-2-naphthoic acid (DHNA) (Bouglé et al., 1999; Kaneko, 1999; Hojo et al., 2002; Ouwehand et al., 2002; Seki et al., 2004; Mitsuyama et al., 2007).

Microorganisms that live in or transit through the digestive tract of humans may establish a symbiotic relationship with the host, thus promoting intestinal homeostasis (de Souza and Fiocchi, 2016). Consumption of *P. freudenreichii* selected strains can enhance human complex intestinal microbiota through the increase of other beneficial bacteria populations, such as bifidobacteria (Bouglé et al., 1999; Kaneko, 1999; Hojo et al., 2002; Ouwehand et al., 2002; Seki et al., 2004; Mitsuyama et al., 2007). In contrast, out of normal physiological conditions, the digestive microbiota may be involved in a variety of immune and inflammatory disorders (Vitetta et al., 2014). One example is inflammatory bowel diseases (IBD), chronic inflammatory disorders that severely affect the digestive tract and may lead, in the long term, to the irreversible deterioration of their structure and function (Belkaid and Hand, 2014; Vitetta et al., 2015). Cheese containing *P. freudenreichii*, in conjunction with *Lactobacillus delbrueckii* (Plé et al., 2016) or as a single strain (Plé et al., 2015), was recently shown to exert immunomodulatory effects, to protect mice against TNBS-induced colitis, to alleviate the severity of symptoms and to modulate local and systemic inflammation markers. Such cheese is currently tested in a pilot clinical trial (ClinicalTrials.gov, 2017). Interestingly, removal of propionibacteria surface-layer (S-layer) proteins, which are non-covalently anchored to the cell surface via an S-layer homology (SLH) domain, suppressed the induction of anti-inflammatory cytokines (Foligné et al., 2010). By contrast, some *P. freudenreichii* strains that possess an extracellular polysaccharide capsule fail to immunomodulate, while mutagenetic suppression of this capsule confers immunomodulatory activity (Deutsch et al., 2012).

Surface proteins of *P. freudenreichii* ITG P20 [*Centre International de Ressources Microbiennes-Bactéries d’Intérêt Alimentaire* (CIRM-BIA) 129], which is used as a cheese ripening starter (Richoux et al., 1998; Thierry et al., 2004), were investigated by a combination of proteomic methods previously developed for bacteria and eukaryotic cells (Lortal et al., 1993; Mayrhofer et al., 2006; Rodríguez-Ortega et al., 2006; Berlec et al., 2011; Bøhle et al., 2011; Bensi et al., 2012; Ythier et al., 2012; Michaux et al., 2013). This investigation demonstrated the involvement of certain S-layer proteins in immunomodulation (Bryson et al., 2006; Le Maréchal et al., 2015). Surface proteins, susceptible to enzymatic shaving and to guanidine extraction, were shown to be involved in the ability of *P. freudenreichii* to modulate the release of cytokines by human immune cells (Le Maréchal et al., 2015). However, the respective role of the different bacterial S-layer proteins was not fully elucidate. Immunomodulation is favored by the ability of specific strains to adhere to the host cells and mucus (Tuomola et al., 1999; Ouwehand et al., 2000; Huang and Adams, 2003; Thiel et al., 2004; Le Maréchal et al., 2015). Indeed, the local action of metabolites and of immunomodulatory molecules is favored by the ability of probiotics to adhere to the host cells. Dairy propionibacteria were shown to adhere to mice intestinal epithelial cells both *ex vivo* and *in vivo* (Zarate, 2012) as well as to cultured human intestinal cell lines *in vitro* (Huang and Adams, 2003; Moussavi and Adams, 2010). However, the precise mechanisms are poorly characterized in *P. freudenreichii*. Adhesion moreover constitutes a key criterion in strain selection and is described as the initial step for colonization of the host (Havenaar et al., 1992, havenar; Riedel et al., 2006; Preising et al., 2010), depending on crucial surface compounds, including surface proteins (Lebeer et al., 2010).

The identification of adhesion mechanisms and molecules is a fundamental step in the elucidation of the bacterium/host cross-talk (van de Guchte et al., 2012). This was lacking in probiotic dairy propionibacteria. The aim of our study was thus to identify *P. freudenreichii* protein(s) involved in adhesion to human intestinal epithelial cells.

## Materials and Methods

### Bacterial Strains and Culture Conditions

The *P. freudenreichii* wild-type (WT) strains, genetically modified strain and plasmids used in this study are listed in **Table 1**. All strains in this study were obtained from the collection of the CIRM-BIA (STLO, INRA Rennes, France). All *P. freuderenichii* WT strains were grown at 30°C in YEL broth (Malik et al., 1968) without agitation or in cow’s milk ultrafiltrate supplemented with 50 mM of sodium L-lactate (galaflowSL60, Société Arnaud, Paris, France) and 5 g/L of casein hydrolysate (Organotechnie, La Courneuve, France), sterilized by 0.2 μm filtration (Nalgene, Roskilde, Denmark) as described previously (Cousin et al., 2012a). For genetically modified strains, YEL and Milk Ultrafiltrate culture media were supplemented with chloramphenicol (10 μg ml^-1^). The growth of *P. freudenreichii* strains was monitored spectrophotometrically by measuring the optical density at 650 nm (OD650), as well as by counting colony-forming units (CFUs) in YEL medium (Malik et al., 1968) containing 1.5% agar. *P. freudenreichii* strains was harvested in a stationary phase (76 h, 10^9^ CFU/mL, determined by plate counts) by centrifugation (6,000 × *g*, 10 min, 4°C). *Escherichia coli* strain DH5α was grown in Luria–Bertani medium at 37°C, and cells carrying DNA plasmid were selected by addition of ampicillin (100 μg ml^-1^).

**Table 1:** *Propionibacterium freudenreichii* wild-type strains, their genetically modified derivatives and plasmids used in the study.

Strains and plasmids	Relevant genotype and phenotype	Source or reference
**Strains *P. freudenreichii*^a^**		
CB 118	Wild-type; SlpA, SlpB, SlpE, and InlA proteins detected in guanidine extract^b^	CIRM-BIA
CB 121	Wild-type; InlA and LspA proteins detected in guanidine extract	CIRM-BIA
CB 129	Wild-type; SlpA, SlpB, SlpE, InlA, and LspA proteins detected in guanidine extract	CIRM-BIA
CB 134	Wild-type; SlpA, SlpE, InlA, and LspA proteins detected in guanidine extract	CIRM-BIA
CB 136	Wild-type; SlpA, SlpB, InlA, and LspA proteins detected in guanidine extract	CIRM-BIA
CB 508	Wild-type; SlpA, SlpB, SlpE, InlA, and LspA proteins detected in guanidine extract	CIRM-BIA
CB 527	Wild-type; Absence of surface layer proteins in guanidine extract	CIRM-BIA
CB 129Δ*slpB*	Cm^r^; CIRM-BIA 129 with chromosomal insertion of pUC:Δ*slpB*:CmR in the *slpB* sequence; SlpB protein absent in guanidine extract	This Study
**Plasmids**		
pUC:slpB	pUC18; Amp; harboring *slpB* partial gene sequence for inactivation	This Study
pUC:ΔslpB:CmR	pUC18 carrying a chloramphenicol resistance gene and harboring *slpB* partial gene sequence	This Study

*^a^CB, CIRM-BIA, Centre International de Ressources Microbiennes–Bactéries d’Intérêt Alimentaire, INRA, UMR 1253, Science et Technologie du Lait et de l’Oeuf, Rennes, France. ^b^Guanidine Hydrochloride treatment used to extract surface layer associated proteins non-covalently bound to the surface is described in “Materials and Methods.”*

### Enzymatic Shaving of Surface Proteins

One hundred microliter of propionibacteria stationary phase culture (see above) were harvested by centrifugation (6,000 × *g*, 10 min, 4°C) and washed in an equal volume of PBS [pH 8.5] containing 5 mM DTT before resuspension in 1/10 volume of the same buffer. Sequencing grade modified trypsin (V5111, Promega, Madison, WI, United States) was dissolved in the same buffer (qsp 0.2 g/L) and added to the bacterial suspension. “Shaving” was performed for 1 h at 37°C in a 0.5 mL reaction volume containing 5 × 10^9^ bacteria and 4 μg of trypsin, with gentle agitation (180 rpm). Bacteria were removed by centrifugation (8,000 × *g*, 10 min, 20°C) and subjected to three washes in PBS prior to adhesion assay.

### Cell Line and Culture Conditions

The human colon adenocarcinoma cell line HT-29 was obtained from ATCC (American Type Culture Collection, Rockville, MD, United States). These cells was cultured under conditions of 37°C, 5% CO_2_, and 90% relative humidity in DMEM High Glucose with L-Glutamine with Sodium Pyruvate (PAN, Dominique Dutscher, Brumath, France) supplemented with 10% heat-inactivated fetal calf serum (FCS) (PAN, Dominique Dutscher, Brumath, France) and antibiotics or not (for adhesion assays).

### Electroporation and Inactivation of the *slpB* Gene in *P. freudenrenichii* CIRM-BIA 129 by Suicide Vector

Inactivation of *P. freudenreichii* gene was adapted from Deutsch et al. (2012) with some modifications. For insertional inactivation of a *slpB* gene, a 520-bp DNA fragment homologous to nucleotides 30–550 of the 5′ region of the slpB coding region in *P. freudenreichii* CIRM-BIA 129 genome was synthesized by Genscript Inc.^1^ with restriction sites *Xba*I-*slpB*-5′ and *BamH*I-*slpB*-3′ resulting in pUC:Δ*slpB* plasmid. The pUC:Δ*slpB* plasmidic DNA was digested with *XbaI* and *BamH*I, purified, and cloned in plasmid pUC:CmR digested by the same enzymes, which resulted in the suicide vector pUC:Δ*slpB*:CmR, which was confirmed by sequencing. See **Supplementary Figure S2**.

Electrocompetent *P. freudenreichii* CIRM-BIA 129 cells was prepared as previously described (Deutsch et al., 2012) with slight modifications. They were cultured in YEL medium supplemented with 0.5 M sucrose and 2% glycine until the early exponential growth phase (OD = 0.1), harvested (6,000 × *g*, 10 min, 4°C). The pellet was washed extensively in ice-cold 0.5 M sucrose and resuspended in electroporation buffer containing 0.5 M sucrose with 10% glycerol and 1 mM potassium acetate (pH 5.5). For electroporation, a 100-μl aliquot of the electrocompetent cells was mixed with 3 μg of pUC:Δ*slpB*:*Cm*R plasmid DNA in a cooled electroporation cuvette. The electroporation of *P. freudenreichii* CIRM-BIA 129 was performed with a Gene Pulser Xcell^TM^ (Bio-Rad Laboratories, Richmond, CA, United States) at 20 kV/cm, 200-Ω resistance, and 25-μF capacitance. Immediately after the pulse, 900 μL of YEL containing 0.5 M sucrose, 20 mM MgCl_2_, and 2 mM CaCl_2_ were added before incubation, 24 H at 30°C under microaerophilic conditions. Cells were plated, and incubated 7 days at 30°C under anaerobic conditions, on YEL medium containing 1.5% agar (YELA) supplemented with 10 μg⋅ml^-1^ of chloramphenicol in order to select *P. freudenreichii* mutants harboring inserted pUC:Δ*slpB*:*Cm*R. The *P. freudenreichii* CIRM-BIA 129 Δ*slpB* (CB129Δ*slpB*) mutant strain was further checked by proteomics for the absence of intact SlpB surface proteins as indicated in the “Results” section. Moreover, the stability of the insertion was checked after three independent cultures in YEL and Milk Ultrafiltrate media without chloramphenicol.

### *In Vitro* Adhesion Assays

Adhesion of *P. freudenreichii* (WT and mutant) to the human colon adenocarcinoma cell line HT-29 was examined by adding 10^8^ live propionibacteria (washed twice in PBS, numerated by CFU conting, ratio 100 bacteria:1 HT-29 cell, MOI 100) to 10^6^ cells in DMEM culture medium without antibiotics. Adhesion assay was conducted by incubation of bacteria/cell at 37°C for 60 min under conditions, 5% CO_2_ and 90% relative humidity. Cells were washed twice with prewarmed PBS pH 7.4, and the subsequently supernatant was removed, and 400 μL of trypsin-EDTA (Invitrogen) was added to each well, before incubation for 5 min at 37°C and to trypsin inactivation by adding 800 μL of DMEM culture medium without antibiotics. Cells were harvested (3,000 × *g*, 3 min) and lysed in 0.1% Triton X-100 before serial dilutions and plating on YELA. Finally, plates were incubated at 30°C for 5 days under anaerobic conditions. A rate of adhesion was calculated as follows: (bacterial count after adhesion experiment/bacterial population added on to HT29 cells). The CIRM-BIA 129 WT strain was then used as a reference in this work, with a % adhesion of 100, and used to normalize all other adhesion rates as a percentage of CIRM-BIA 129 WT adhesion. Each adhesion assay was conducted in technical and biological triplicates. To test involvement of surface proteins in adhesion, propionibacteria were subjected (or not) to enzymatic shaving (see section “Enzymatic Shaving of Surface Proteins”) before adhesion assay. To confirm this hypothesis, propionibacteria were incubated 60 min at 37°C with 50 μg of *P. freudenreichii* CIRM-BIA 129 guanidine-extracted S-layer associated proteins, in solution in PBS, under agitation, before adhesion. This amount (50 μg) was determined after preliminary experiments to determine amounts efficient in restoring adhesion. For specific inhibition of adhesion by antibodies directed against SlpB, propionibacteria were incubated in PBS pH 7.4 with immunoglobulins purified from rabbit anti-SlpB serum (AGRO-BIO, France) in 1:10.000 dilution, under agitation, 60 min at 37°C. Propionibacteria were washed twice with PBS pH 7.4 before adhesion assay.

The adhesion ratio of CB 129 strain alone was used as a reference to calculate the adhesion rates of different strains and treatments.

Internalization of bacteria was determined as previously described (Bouchard et al., 2013) 2-h post contact following an additional 2-h incubation step with DMEM supplemented with gentamicin (100 μg/ml) to kill extracellular bacteria. Subsequently, HT-29 cells monolayers were washed three times with PBS, treated with trypsin, centrifuged for 5 min at 800 × *g*, and lysed in 0.01% Triton to allow the numeration of internalized propionibacteria population only.

### Bacterial Cell Adhesion Observation by Microscopy

Observation of *P. freudenreichii* adhesion to cultured human colon epithelial cells was as described previously for lactobacilli (Tiptiri-Kourpeti et al., 2016), with modifications for propionibacteria. Briefly, propionibacteria, cultured as described above, were washed and resuspended in PBS, prior to the addition of 20 μM CFSE (carboxyfluorescein succinimidyl ester, cell trace proliferation kit for flow cytometry ref C34554 Thermo Fisher Scientific, Waltman, MA, United States), freshly prepared as a 1,000× solution in DMSO and kept in the dark. Incorporation of CFSE was allowed for 30 min at 30°C in the dark, prior to washing and resuspension of propionibacteria in YEL medium, 30°C. Hydrolysis of CFSE by intracellular esterase activity was allowed 30 min at 30°C in the dark to generate intracellular fluorescence. Fluorescence was checked on an epifluorescence microscope (BX-51, Olympus, equipped with a U-MWB2 fluorescence filter cube). HT-29 cells were cultured in Lab-Tek chamber slide (Thermo Fisher Scientific) and labeled bacteria were added to a 1/100 ratio (1 × 10^6^ cells, 1 × 10^8^ bacteria, in 1 mL of DMEM) before incubation, 1 h, 37°C. After two washes with PBS, the plasma membrane of cells was then labeled with the exogenous head-labeled phospholipid fluorescent probe *N*-(Lissamine rhodamine B sulfonyl) dioleoyl phosphatidylethanolamine (Rh-DOPE, Avanti Polar Lipids Inc., Birmingham, England) used at a concentration of 1 μg/mL in DMEM, for 10 min. Cell layer was then washed twice with PBS and slides mounted in DAPI (4,6-diamidino-2-phenylindole)-containing mounting medium (Vectashield mounting medium for fluorescence Vector ref *H-1200*). Cells were observed using a confocal Leica SP8 and a 63/1.4 oilHC PL APO CS objective. Images were acquired using LAS-AF (Leica, Wetzlar, Germany) software.

For scanning electron microscopy, HT-29 cells were cultured in Corning^®^ Transwell^®^ polycarbonate membrane cell culture inserts on polycarbonate 0.4 μm pore size filtration membrane. Adhesion was conducted as described above. Membranes were then removed, washed in PBS, fixed 48 h by 2% (wt/vol) glutaraldehyde in 0.1 M sodium cacodylate buffer [pH 6.8] and rinsed in the same buffer. Samples were dehydrated with ethanol (10, 25, 50, 75, 95, and finally 100%), critical-point dried by the CO_2_ method and coated with gold. Cells were examined and photographed with a Philips XL 20 scanning electron microscope operating at 10 kV.

### Bacterial Cell Adhesion Determination by Cytometric Analysis

Determination of *P. freudenreichii* adhesion to cultured human colon epithelial cells was performed as described previously for lactobacilli (Tiptiri-Kourpeti et al., 2016). Cells were cultured in DMEM as described above to confluence. CFSE-labeled bacteria were added as described above before a 1-h incubation at 37°C. Cells were trypsinized and analyzed by fluorescence cytometry using an excitation wavelength of 488 and emission at 585 nm (Accuri C6 Becton Dickinson, Le Pont-de-Claix, France). Data were collected from 50,000 cells and analysis was performed with CFlow software.

### Guanidine Extraction of Surface Layer Associated Proteins Non-covalently Bound to the Cell Wall

Propionibacteria cultures in stationary phase (76-h) were collected by centrifugation (8,000 × *g*, 10 min, 4°C) for extraction of S-layer proteins by Guanidine Hydrochloride (GuaHCl) (Le Maréchal et al., 2015). The bacterial pellet was washed two times with an equal volume of PBS buffer pH 7.4. This pellet was resuspended in 5 M GuaHCl to a final OD_650_ of 20 then incubated 15 min at 50°C, and subsequently, the suspension was centrifuged (21.000 × *g*, 20 min, 30°C). The cells were eliminated, and the supernatant was dialyzed extensively against PBS buffer pH 7.4 (for adhesion assays) or 0.1% SDS (for SDS–PAGE analysis) for 24 h at 4°C using Slide-A-Lyer^®^ Dialysis Cassette (ThermoScientific, Rockford, IL, United States). This procedure was applied in three independent cultures.

### One-Dimensional SDS–Polyacrylamide Gel Electrophoresis (1-DE) and Western Blotting

Extracts of S-layer proteins in 0.1% SDS were diluted in SDS sample buffer and then heat-denatured 10 min at 95°C. One-dimensional polyacrylamide gel electrophoresis (10.0%) was conducted according to Laemmli (Laemmli, 1970) on a Mini-PROTEAN^®^ Tetra Cell (Bio-Rad, Hercules, CA, United States) and the gels were stained using Coomassie Blue Bio-Safe reagent (Bio-Rad). Alternatively, S-layer protein associated extracts were separated by 10% SDS–PAGE and transferred to PVDF membranes (GE Healthcare). After blocking with 3% non-fat dry milk diluted in TBS (Tris 10 mM, NaCl 0.15 M, 0.3% tween 20), the membranes were incubated overnight at 4°C with primary antibodies purified from rabbit sera (AGRO-BIO, France). These were obtained by injecting the following slpB peptide to rabbits: IDATVDKQNSKGGFGWGG and used at the dilution 1:10,000. After washing, membranes were incubated with secondary antibodies: anti-rabbit IgG conjugated with horseradish peroxidase (1:15,000, AGRO-BIO, France) for 2 h at room temperature. Bound antibodies were visualized with ECL Plus system (GE Healthcare, Vélizy, France) and blots were scanned using the Syngene GBox (Ozyme, Saint-Quentin-en-Yvelines, France). The specificity of anti-SlpB western blotting was checked (**Supplementary Figure S1**). A single band was observed only in strains expressing SlpB and the labeling pattern was distinct from that of anti-SlpA and anti-SlpE western blotting.

### Data Analysis

All the experiments were performed with three technical replicates and three biological replicates, and the results were expressed as means ± standard deviations (SD). Statistical analyses were performed in R Statistical Software (Foundation for Statistical Computing, Vienna, Austria) using ANOVA with Tukey *post hoc* analyses for multiple comparisons.

## Results

### Surface Layer Associated Proteins and Adhesion to Cultured Human Colon Cells Are Variable among Strainsof *P. freudenreichii*

**FIGURE 1:**
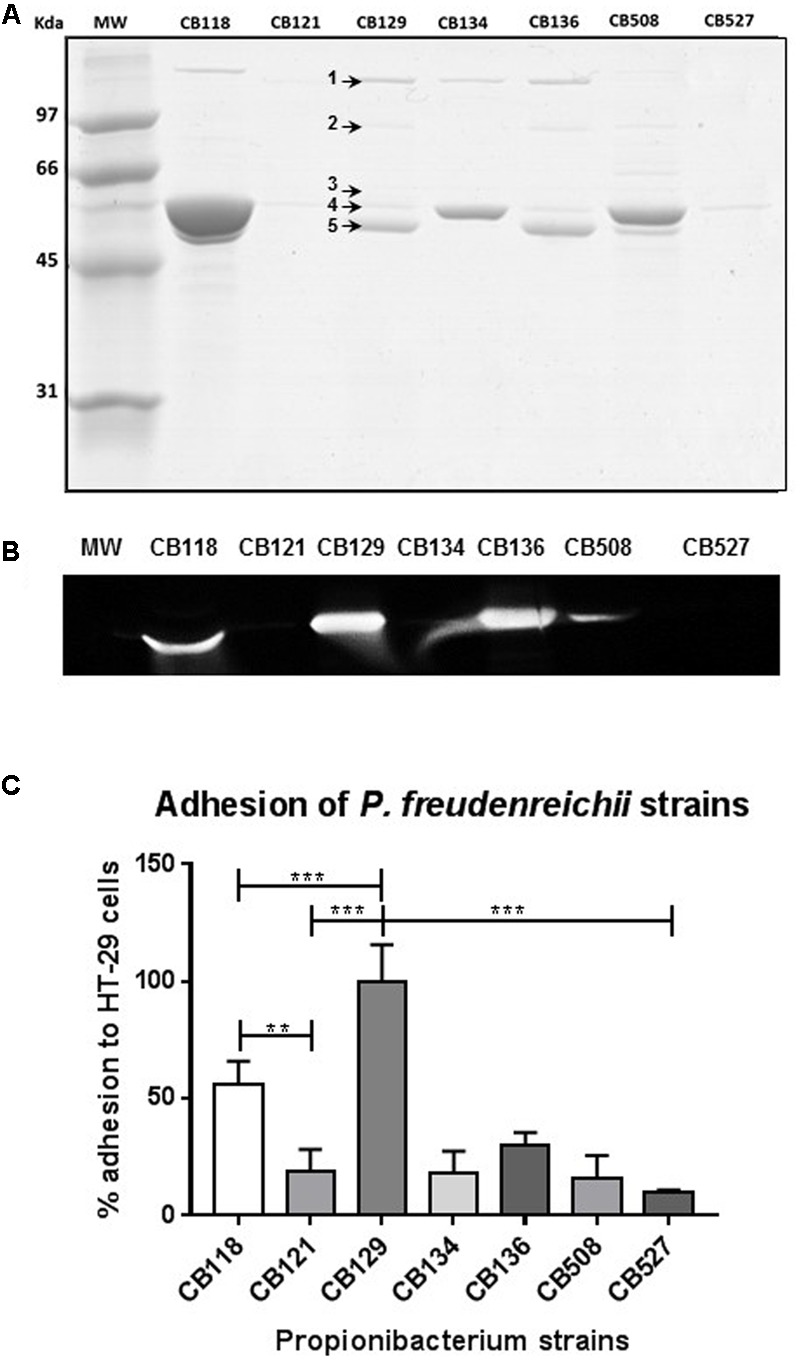
Variability of surface proteome and of adhesion among strains of *Propionibacterium freudenreichii*. **(A)** Guanidine-extracted surface layer associated proteins are variable. Seven strains of *P. freudenreichii* were cultured in milk ultrafiltrate and subjected to guanidine-extraction followed by SDS–PAGE (10%) gel electrophoretic analysis of the extracts. Gels were either Coomassie-Blue-stained **(A)** or transferred to a PVDF membrane. Surface proteins previously identified by mass spectrometry as InlA, LspA, SlpE, SlpA and SlpB in strain CB129 are indicated by 1, 2, 3, 4, and 5, respectively. **(B)** Western Blotting detection of surface layer protein SlpB. PVDF membranes were treated using rabbit antibodies raised against *P. freudenreichii* surface layer protein SlpB. **(C)** Adhesion to cultured human colon epithelial cells is variable. HT-29 cells were cultured to confluence in DMEM prior to co-incubation. Each well (1 × 10^6^ HT-29 cells) was added with 1 × 10^8^ colony-forming unit (CFU) of *P. freudenreichii*. Co-incubation was 60 min at 37°C in DMEM. After thorough washing with PBS, adhered bacteria were enumerated by CFU plate counting in trypsinized cells. Numbers of the strains used are indicated. Asterisks represent statistically significant differences between strains and were indicated as follows: ^∗^*p* < 0.05; ^∗∗^*p* < 0.01; ^∗∗∗^*p* < 0.001. Adhesion is presented as a percent of the reference CB129 *P. freudenreichii* strain. Original gels and western blots, uncropped, are provided in **Supplementary Figure S1**.

Seven strains of *P. freudenreichii* from the CIRM-BIA collection (**Table 1**), CB 118, CB 121, CB 129, CB 134, CB 136, CB 508, and CB 527, have been selected based on preliminary proteomic screening as they all displayed different surface proteomes as shown by their S-layer associated protein pattern after guanidine treatment (**Figure 1A**). The five proteins, previously identified in CB 129 (SlpA, SlpB, SlpE, InlA, and LspA, see Le Maréchal et al., 2015), and thought to play a role in interactions with the host, are indicated in the figure. Preliminary results pointed out SlpB as a potential key surface protein in *P. freudenreichii.* We thus developped antibodies in order to confirm this. Western blot analysis using these antibodies further confirmed variability of surface proteins (**Figure 1B**). SlpB was detected in four strains out of seven, with different intensities. The variability of S-layer associated proteins suggested possible variations regarding interactions with host cells. The seven strains were further compared with respect to adhesion to HT-29 cultured colon cells (**Figure 1C**). The CB129 strain, exhibited the highest adhesion rate (6.44 CFU/1 HT-29 cell) and was used as the reference (100% adhesion) for comparison with the other strains (100.0% ± 17). Indeed, CB129 showed a significant difference (*p* < 0.001) with the other *P. freudenreichii* strains tested under the same experimental conditions. The CB118 strain exhibited a lower but significant adherence percentage of 56.0% ± 10.0 and also displayed SlpB. All the other strains exhibited low adhesion rates without significant differences among them, although CB136 (30.0% ± 5.0), which also displays SlpB, tended to be more adhesive than the rest of this subset. Finally, the lowest adhesion rate was recorded for CB527, 10.0% ± 1.0, for which no surface protein was detected, in accordance with (Deutsch et al., 2017). Different propionibacteria: HT-29-cells ratios were tested for adhesion (100:1, 500:1, and 1,000:1, in technical and biological triplicates) with similar results in adhesion rates ranking. At the MOI of 100:1 used in this study, no internalization of *P. freudenreichii* was observed (data not shown) using the gentamicin method used by our team to monitor staphylococci internalization (Bouchard et al., 2013).

### *P. freudenreichii* CB129 Interacts with Cultured Human Colon Cells

**FIGURE 2:**
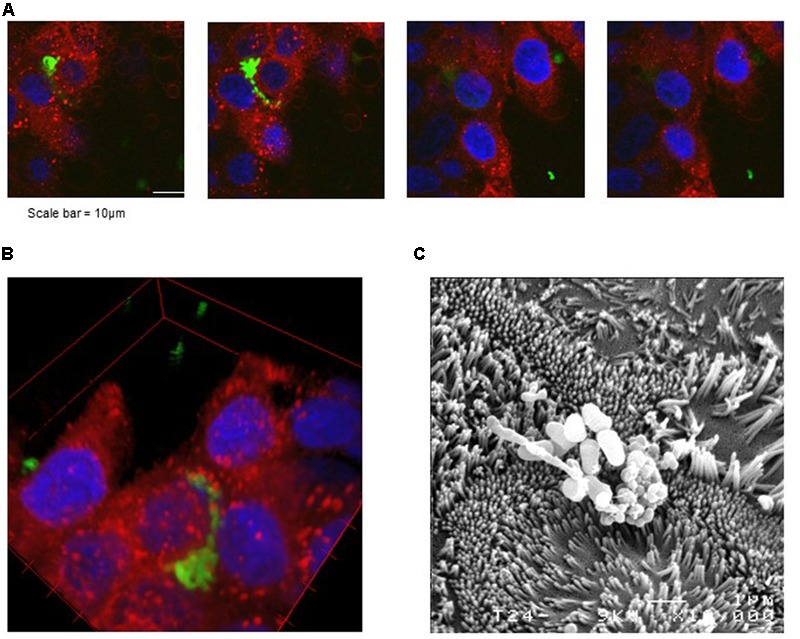
Microscopy imaging of *P. freudenreichii Centre International de Ressources Microbiennes–Bactéries d’Intérêt Alimentaire* (CIRM-BIA) 129 adhesion to cultured human colon epithelial cells. HT-29 cells were cultured to confluence in DMEM on a slide chamber prior to interaction. *P. freudenreichii* was cultured in fermented milk ultrafiltrate prior to intracellular labeling of live bacteria using CFSE. Labeled bacteria were then co-incubated with colon cells in a slide chamber prior to washing with PBS and to staining of plasma membrane using Rh-DOPE and mounting in DAPI-containing mounting medium. **(A,B)** Blue fluorescence evidences colon cells nuclei, red fluorescence their plasma membrane and green fluorescence CFSE-labeled propionibacteria. **(A)** Z-stack, i.e., confocal images acquired at different “z” altitudes in the labeled preparation. **(B)** Reconstituted 3-D image showing a cluster of propionibacteria at surface of cells. **(C)** Scanning electron microscopy observation of propionibacteria adhesion to cultured colon epithelial cells. The same co-incubation was performed in a polycarbonate membrane cell culture insert prior to fixation and scanning electron microscopy observation.

Adhesion of *P. freudenreichii* to HT-29 cells being demonstrated, we further looked at such an interaction, using three-dimensional confocal microscopy. As seen in **Figure 2A**, the sections close to the bottom of the slide culture chamber mainly exhibited the blue fluorescence of the HT-29 nuclei, stained with DAPI, a poorly fluorescent cytoplasm, surrounded by a red-stained plasma membrane (lowest images in **Figure 2A**). Ascending within this “z-stack,” higher sections showed dots with intense red fluorescence, corresponding to cell membranes, indicative of colonocytes microvilli constituting the brush border. Higher sections showed co-localization of these red dots with green-fluorescent propionibacteria, caused by CFSE metabolization within propionibacteria. More precisely, propionibacteria appeared as aggregates, in the intercellular space of the epithelial HT-29 monolayer. This localization of propionibacteria in contact with cells is further illustrated in the reconstituted 3-D view (**Figure 2B**). Interaction of propionibacteria with cultured human colonocytes was further illustrated by scanning electron microscopy of co-cultures on cell culture inserts (**Figure 2C**). This revealed localization of propionibacteria at the surface of cells, in contact with the brush border.

### *P. freudenreichii* CB129 Adhesion to Cultured Human Colon Cells Involves Surface Proteins

**FIGURE 3:**
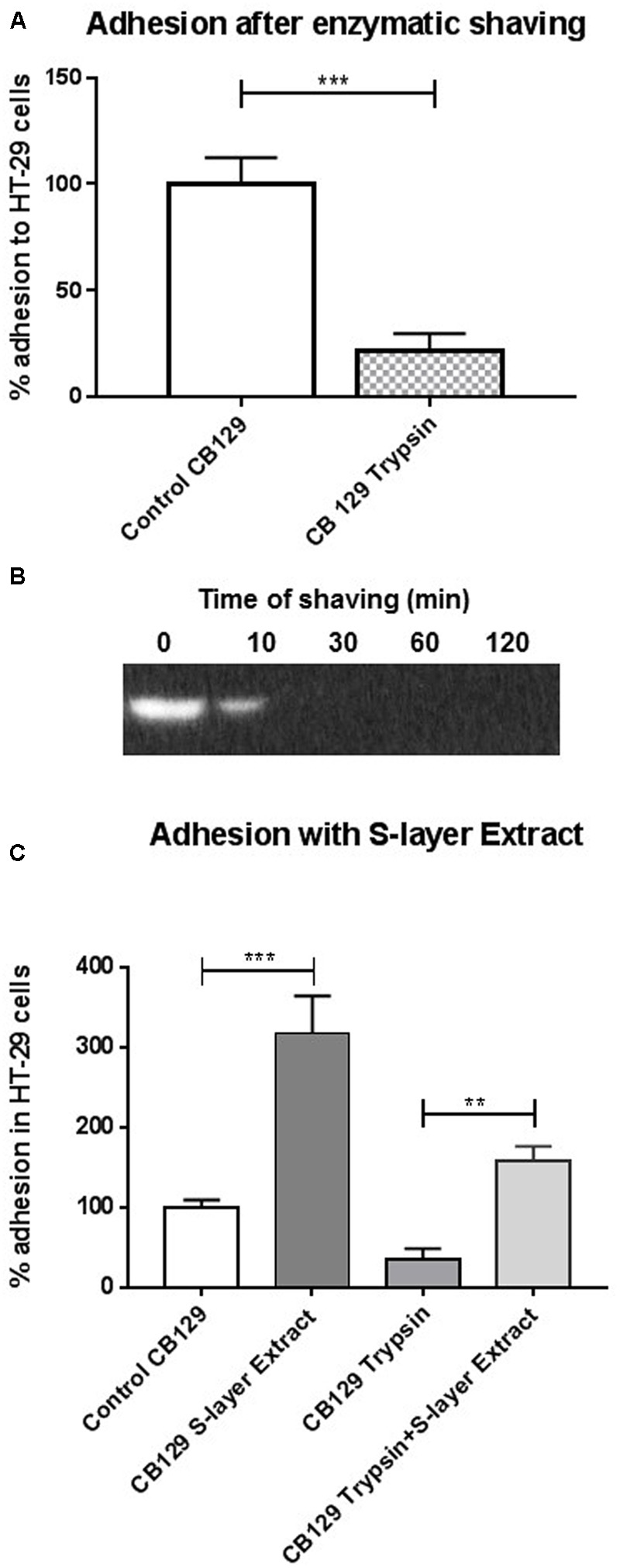
Involvement of *P. freudenreichii* surface proteins in adhesion. **(A)** Trypsin shaving reduces *P. freudenreichii* CIRM-BIA 129 adhesion. Human colon cells were cultured in DMEM prior to co-incubation with propionibacteria. Used propionibacteria were either untreated (control) or submitted to trypsin shaving of surface proteins for 60 min (trypsin). Adhered bacteria were enumerated by CFU plate counting in trypsinized cells. **(B)** Trypsin shaving reduces presence of SlpB protein in *P. freudenreichii* CIRM-BIA 129 in different times of incubation. *P. freudenreichii* CIRM-BIA 129 show after different incubations time with trypsin (T_zero_ min, T_30_ min, T_60_ min, and T_120_ min) a decreased amount of SlpB in Western Blot analysis with anti-SlpB antibodies. **(C)** Addition of extracted surface layer proteins enhances *P. freudenreichii* CIRM-BIA 129 adhesion. Human colon cells were cultured prior to co-incubation with propionibacteria. Used propionibacteria were either shaved for 60 min (trypsin) or untreated (control). They were then added with surface layer guanidine extract (50 μg of proteins) or not. Adhesion was quantified by plate CFU counting of propionibacteria after trypsinization of colon cells. Adhesion is presented as a percent of the reference CIRM-BIA 129 *P. freudenreichii* strain. Asterisks represent statistically significant differences between strains and were indicated as follows: ^∗^*p* < 0.05; ^∗∗^*p* < 0.01; ^∗∗∗^*p* < 0.001. Adhesion is presented as a percent of the reference CB129 *P. freudenreichii* strain.

To determine whether the presence of surface proteins is involved in the adhesion of *P. freudenreichii* to HT-29 cells, the method of enzymatic shaving using trypsin was applied, before adhesion assay. A significant reduction (*p* < 0.001) was observed in the adhesion rate: 21.77 ± 8.10% for shaved bacteria, compared to the positive control consisting of propionibacteria (**Figure 3A**). Western blot analysis also indicated absence of SlpB at the surface of *P. freudenreichii* as a result of shaving (**Figure 3B**). To further confirm the role of surface proteins in adhesion, *P. freudenreichii* CB129 cells, shaved or not, were incubated with 50 μg of extracted surface proteins. This guanidine extract from the CB129 strain was previously dialyzed against PBS and quantified by Bradford assay. It contained the five proteins (SlpA, SlpB, SlpE, InlA, and LspA, see **Figure 1A**) in PBS buffer pH 7.4. Adhesion assay was then conducted. This incubation increased the rate of adhesion of *P. freudenreichii* CB129 to HT-29 cells, from 100.00% ± 8.93 to 317.07% ± 46.68. Furthermore, adhesion rate, which was strongly diminished by enzymatic shaving (33.99% ± 14.30), was restored by this incubation (157.44% ± 18.31, **Figure 3C**). This further experiment confirmed the key role of at least one of these surface proteins in adhesion.

### Surface Protein SlpB Plays a Key Role in Adhesion to Cultured Human Colon Cells

**FIGURE 4:**
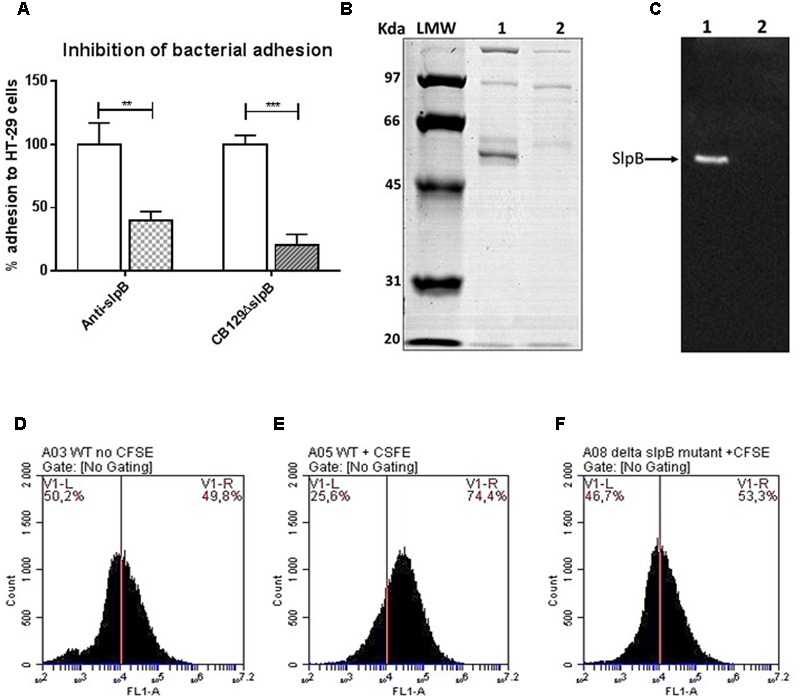
Key role of surface layer protein SlpB in adhesion. **(A)** Antibodies reduce *P. freudenreichii* adhesion and so does *slpB* gene inactivation. Human colon cells were cultured in DMEM prior to co-incubation with propionibacteria. *P. freudenreichii* CIRM-BIA 129 was treated with antibodies raised against SlpB prior to adhesion assay. As an alternative, the *slpB* gene was inactivated in *P. freudenreichii* CIRM-BIA 129 prior to adhesion assay. Adhesion is presented as a percent of the reference CB129 *P. freudenreichii* strain. **(B)** Guanidine-extracted surface layer associated proteins were compared by SDS–PAGE in *P. freudenreichii* CIRM-BIA 129 wild-type (line 1) and in the corresponding mutant CB129*ΔslpB* (line 2). **(C)** The corresponding PVDF membrane was subjected to Western Blotting using rabbit antibodies raised against *P. freudenreichii* surface layer protein SlpB **(B)**. **(D,E)** Fluorescently labeled live *P. freudenreichii* CIRM-BIA 129 adheres to cultured human colon epithelial HT-29 cells. The adhesion of CFSE-labeled propionibacteria was detected by the shift in FL1 intensity **(E)**, compared to HT-29 cells with unlabelled propionibacteria **(D)**. Cells (10^6^) were co-incubated with 10^8^ CFU of CFSE-stained propionibacteria for 1 h. At least 50.000 cells per sample were analyzed. As an alternative, labeled *P. freudenreichii* mutant CB129*ΔslpB* was co-incubated with HT-29 cells **(F)**. Original gels and western blots, uncropped, are provided in **Supplementary Figure S1**. Asterisks represent statistically significant differences between strains and were indicated as follows: ^∗^*p* < 0.05; ^∗∗^*p* < 0.01; ^∗∗∗^*p* < 0.001. Adhesion is presented as a percent of the reference CB129 *P. freudenreichii* strain.

In a second approach to inhibit adhesion and to precise the role of specific surface proteins, *P. freudenreichii* was incubated with antibodies raised against SlpB, at a dilution of 1:10,000, before adhesion assay. This resulted in a significant reduction following incubation with the anti-SlpB antibodies 39.95% ± 6.92 (*p* < 0.001), (**Figure 4A**). We then further focused on SlpB and inactivated its gene in *P. freudenreichii* CB129. The mutant *P. freudenreichii* CB129Δ*slpB* was obtained by insertion of the pUC:Δ*slpB*:*Cm*R suicide plasmid as described in the “Materials and Methods” section (**Supplementary Figure S2**). The stability of the mutant was validated after growth in the presence or absence of chloramphenicol by checking for the absence of SlpB production. As shown in **Figure 4B**, one protein band (about 55 kDa in size) was lacking in the mutant S-layer associated proteins guanidine extract (line 2), when compared to the WT parental strain (line 1). Western Blot analysis using antibodies raised against the SlpB protein (**Figure 4C**) confirmed that this protein was effectively mutated in the mutant (line 2) when compared to the parental strain (line 1). Efficient and specific inactivation of the *slpB* gene was further established by mass spectrometry analysis of guanidine-extracted S-layer proteins. Indeed, the SlpA, SlpB, and SlpE proteins were clearly identified in the WT CB129 strains, while only SlpA and SlpE were detected in the mutant *P. freudenreichii* CB129Δ*slpB* strain (**Table 2**).

**Table 2:** Surface-layer proteins identified after Guanidine Hydrochloride extraction.

		Wild-type strain			Delta SlpB strain		
Gene name	Locus tag	Log (*e*-value)^a^	Cover (%)^b^	Peptides^c^	Log (*e*-value)^a^	Cover (%)^b^	Peptides^c^
SlpB	PFCIRM129_00700	-263.2	74	34	0	0	0
SlpE	PFCIRM129_05460	-125.6	50	19	-138.8	55	18
SlpA	PFCIRM129_09350	-174.3	75	24	-143.5	68	22

*^a^The *e*-value is the probability that a given peptide score will be achieved by incorrect matches from a database search. Protein *e*-value is the product of individual peptide *e*-value. Protein identifications were automatically validated when they showed at least two unique peptides with an *e*-value below 0.05 corresponding to log (*e*-value) < -1.3. ^b^The percentage of the protein amino acid sequence covered by tandem mass spectrometry identification of peptides. ^c^Number of unique peptide sequence identified with an individual *e*-value < 0.01 for this protein.*

Adhesion to HT-29 cells was then assessed by CFU counting and the mutant CB129Δ*slpB* strain was impaired in adhesion (20.66% ± 8.32) when compared to the WT control (100.00% ± 7.37) (**Figure 4A**, *p* < 0.001). To confirm this result, adhesion of *P. freudenreichii* to HT-29 cells, using CFSE-stained propionibacteria, was quantified by flow cytometry. Cells were treated with CFSE-labeled propionibacteria, WT or CB129Δ*slpB* mutant, for 1 h, before cytometric monitoring of cell fluorescence (**Figures 4D–F**). A shift in fluorescence intensity (FL1) was observed as a result of fluorescent *P. freudenreichii* CB129 adhesion to cells (**Figure 4E**) when compared with control cells without bacteria (**Figure 4D**). This indicates an increase of fluorescence emission at 488 nm, corresponding to 6-carboxyfluorescein succinimidyl harbored by adhering bacteria, as described previously for lactobacilli (Tiptiri-Kourpeti et al., 2016). By contrast, the mutant CB129Δ*slpB* strain failed to reproduce this fluorescence shift in HT-29 cells, and the pattern (**Figure 4F**) was similar to that of HT-29 without bacteria (**Figure 4A**). Altogether, these results confirm the key role of the SlpB surface protein in adhesion of *P. freudenreichii* to HT-29 cells.

## Discussion

Adhesion is a key determinant of host/bacterium interactions, whether pathogenic or probiotic. Adhesion of probiotic bacteria to host intestinal cells may favor important effects including modulation of mucus secretion (Mack et al., 2003), of defensin production (Schlee et al., 2007, 2008), or the local action of beneficial metabolites. It can improve competitive exclusion of pathogens by adhesion competition (Servin, 2004; Lebeer et al., 2008) and constitutes a key factor for several clinical applications of probiotics in the prevention and treatment of gastrointestinal disorders and of IBD. It may involve, on the bacterial side, various microorganism-associated molecular patterns (MAMPs) including flagellin, fimbriae (also called pili) or other surface proteins including moonlighting proteins and S-layer proteins (Lebeer et al., 2010).

Surface-layer proteins constitute a field of research that deserves further investigation. Although anchored to the cell wall via conserved SLH domains, their extracellular protruding part is highly variable, poorly conserved amongst bacterial species and strains. A previous paradigm described S-layers as a macromolecular paracrystalline network formed by the self-assembly of numerous copies of one monomeric protein or glycoprotein and constituting an extracellular S-layer in many bacteria (Sleytr, 1997; Sára and Sleytr, 2000). This was later challenged by studies on *Lactobacillus acidophilus* showing that a S-layer can contain various S-layer proteins or SLPs (Hymes et al., 2016). These proteins are in fact versatile molecules that may play an important role in growth and survival, maintenance of cell integrity, enzyme display, molecular sieving, co-aggregation, immunomodulation, as well as adhesion and persistence within the animal host (Lebeer et al., 2010; Fagan and Fairweather, 2014). In *P. freudenreichii*, such proteins were shown to be involved in immunomodulatory interactions with the host (Le Maréchal et al., 2015), a property highly strain-dependent (Mitsuyama et al., 2007; Foligné et al., 2010, 2013). Indeed, a functional role in immunomodulation by *P. freudenreichii* was recently attributed to a set of proteins: SlpB, SlpE, two putative S-layer proteins with SLH domains, and HsdM3, predicted as cytoplasmic (Deutsch et al., 2017).

Variability of *P. freudenreichii* surface proteins may thus be related to variability in functional properties. In this context, we have selected in the present work seven *P. freudenreichii* strains with different patterns evidenced in a preliminary study.

We confirm here that *P. freudenreichii* S-layer proteins are variable, and so is its ability to adhere to cultured human epithelial cells, as determined by quantitative culturing (Mack et al., 1999), which suggests a functional link between variations in the surface protein pattern. *P. freudenreichii* CIRM-BIA 129, shown to alleviate symptoms of acute colitis in mice, displays S-layer associated proteins and the highest adhesion ability, whatever the bacteria/cell ratio (100:1; 500:1; and 1,000:1). Moreover, at a ratio of 100/1, no internalization was observed. This suggests that propionibacteria either do not internalize into cultured HT-29 cells, or do not suvive within the cells. Cultured colon epithelial HT-29 cells do not produce mucus in our conditions. This suggests that *P. freudenreichii* interacts with epithelial cell surface compounds rather than mucins, a property previously reported for the probiotic *L. acidophilus* (Johnson et al., 2013). Interestingly, CB129 was shown to restore expression of ZO-1, a key protein of tight junctions which expression was impaired in colitis (Plé et al., 2015), as part of its anti-inflammatory effect. Adhesion close to these junctions may favor the local action of propionibacteria via local release of propionate, the major metabolite of propionibacteria, which was shown to improve intestinal barrier function and to restore expression of ZO-1 in DSS-treated mice (Tong et al., 2016). Accordingly, protection toward inflammation-induced barrier defects was reported for the probiotic product VSL#3 (Krishnan et al., 2016).

Enzymatic shaving of surface proteins reduced adhesion and was previously shown to hydrolyze at least 16 distinct proteins (Le Maréchal et al., 2015). Dramatic inhibition of adhesion was observed following blockage with antibodies raised against SlpB. Interruption of the *slpB* gene in CB129 strain also resulted in a drastic reduction (*P* < 0.01) in adhesion. Moreover, addition of purified S-layer proteins restored the adhesion that was suppressed in *P. freudenreichii* by enzymatic shaving. Altogether, these results indicate a role of *P. freudenreichii* S-layer protein, including SlpB, in adhesion, as was reported for the SlpA protein in *L. acidophilus* NCFM (Buck et al., 2005).

This study evidenced a key role of one of the *P. freudenreichii* S-layer proteins in adhesion to human intestinal cells. Understanding determinants of probiotic action is a key challenge. It opens new avenues for the screening of most promising propionibacteria strains, by monitoring their expression, and for the development of new functional products containing them. It is particularly relevant in the context of pathogens competitive exclusion and inflammation remediation.

## Author Contributions

GJ and FdC designed the research. GJ, YL, and VA supervised the work. FdC, HR, SH, FG, MD, SD, and JJ took part to the experiments. FdC and GJ wrote the manuscript. YL and VA corrected the manuscript.

## Conflict of Interest Statement

The authors declare that the research was conducted in the absence of any commercial or financial relationships that could be construed as a potential conflict of interest.

## Footnotes

1 www.genscript.com

## Supplementary Material

FIGURE S1 Specificity of the anti-SlpB antibodies. The whole gels (I, II) and whole blots (II, IV) corresponding to **Figures 1, 4** are shown. A single band reacting with anti-SlpB antibodies is evidenced. Moreover, specific inactivation of slpB gene leads to disappearance of this reactive band (IV). Finally, western blot using anti-SlpB antibodies reveals the SlpB protein only in strains which harbor the corresponding slpB gene, as indicated by the Table (V). In supplemental western blots of the same extracts (VI), sera directed against SlpA and SlpE evidence a distinct pattern. In particular, the two close Coomassie-stained bands, 58 and 56 kDa, were identified by western blot (this work) and by mass spectrometry (Le Maréchal et al., 2015) as SlpA and slpB, respectively.

FIGURE S2 Interruption of *slpB* gene using suicide vector pUC:Δ*slpB*:*CmR*. **(A–C)** Schematic view of homologous recombination producing a mutant CB 128Δ*slpB*. Disruption of *slpB* gene in CB 129 WT by suicide vector pUC:*CmR* harboring 520-bp of *slpB*. Mutant strain show a chloramphenicol resistance by insertion of cassette containing *CmR*. **(D)** Targeting sequence used to inactivate. Partial sequence of *slpB* gene in CB 129 WT and sequence used to homologous recombination (red). The primers annealing site are indicated as underlined bases and oligonucleotides sequence are shown in figure.
