# Supplementary figures and images for: Propionibacterium freudenreichii Surface Protein SlpB Is Involved in Adhesion to Intestinal HT-29 Cells
Source: Front Microbiol. 2017 Jun 8;8:1033. doi: 10.3389/fmicb.2017.01033 (PMC5462946; doi:10.3389/fmicb.2017.01033)

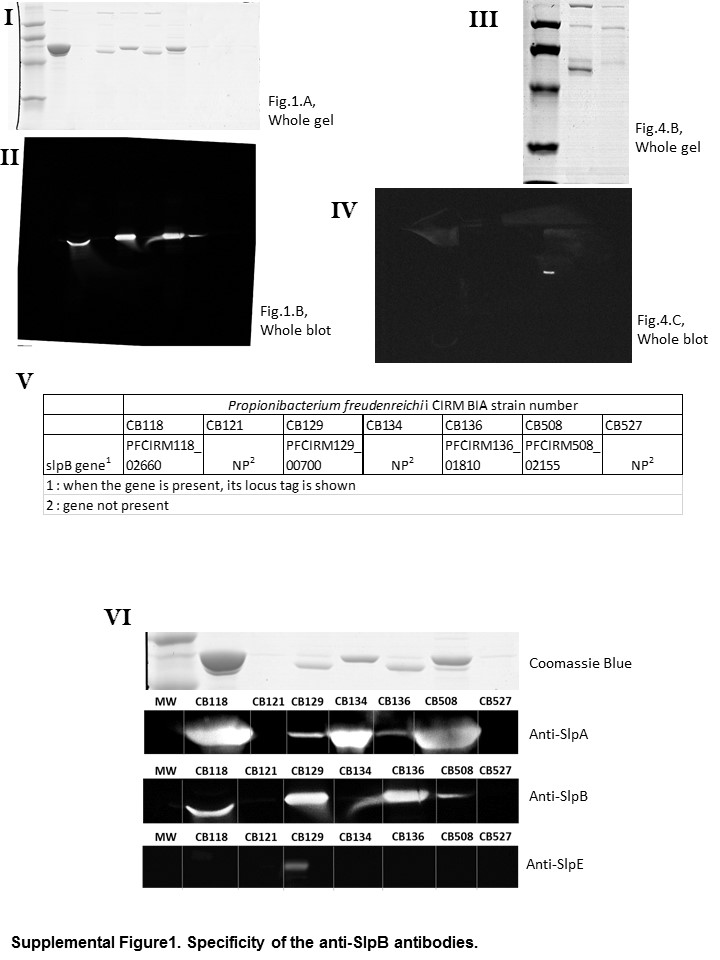

Supplement: FIGURE S1 — Specificity of the anti-SlpB antibodies. The whole gels (I, II) and whole blots (II, IV) corresponding to Figures 1, 4 are shown. A single band reacting with anti-SlpB antibodies is evidenced. Moreover, specific inactivation of slpB gene leads to disappearance of this reactive band (IV). Finally, western blot using anti-SlpB antibodies reveals the SlpB protein only in strains which harbor the corresponding slpB gene, as indicated by the Table (V). In supplemental western blots of the same extracts (VI), sera directed against SlpA and SlpE evidence a distinct pattern. In particular, the two close Coomassie-stained bands, 58 and 56 kDa, were identified by western blot (this work) and by mass spectrometry (Le Maréchal et al., 2015) as SlpA and slpB, respectively. [file Image_1.jpg]

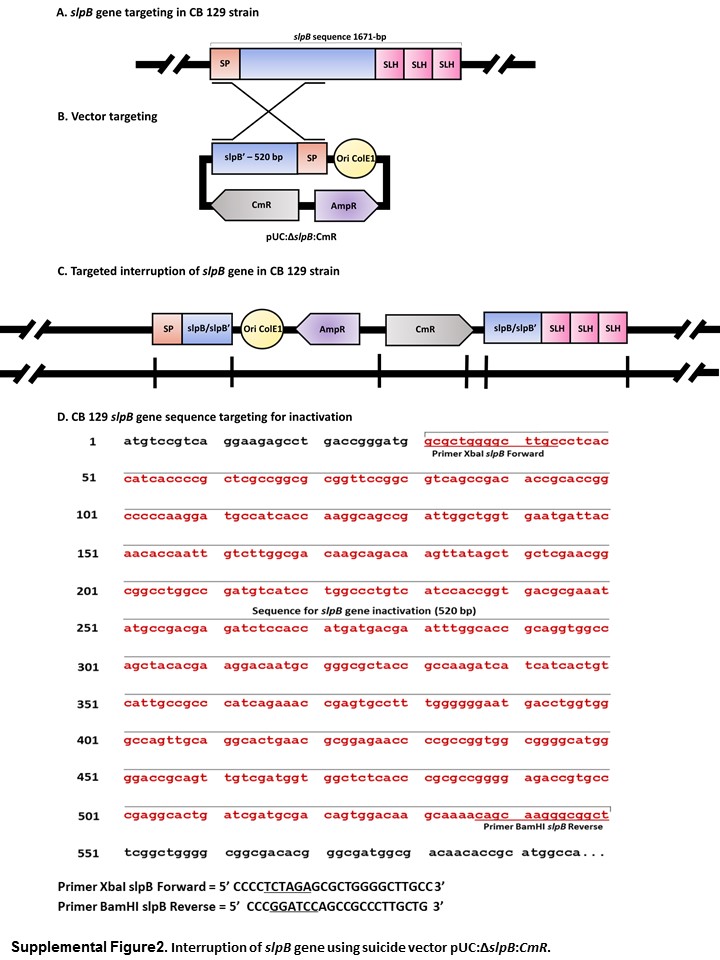

Supplement: FIGURE S2 — Interruption of slpB gene using suicide vector pUC:ΔslpB:CmR. (A–C) Schematic view of homologous recombination producing a mutant CB 128ΔslpB. Disruption of slpB gene in CB 129 WT by suicide vector pUC:CmR harboring 520-bp of slpB. Mutant strain show a chloramphenicol resistance by insertion of cassette containing CmR. (D) Targeting sequence used to inactivate. Partial sequence of slpB gene in CB 129 WT and sequence used to homologous recombination (red). The primers annealing site are indicated as underlined bases and oligonucleotides sequence are shown in figure. [file Image_2.jpg]
